# Core components of clinical education: a qualitative study with attending physicians and their residents

**Published:** 2016-04

**Authors:** ALIREZA ESTEGHAMATI, HAMIDREZA BARADARAN, ALIREZA MONAJEMI, HAMID REZA KHANKEH, MEHRNAZ GERANMAYEH

**Affiliations:** 1 Department of Internal Medicine, Faculty of Medicine, Tehran University of Medical Sciences, Tehran, Iran;; 2 Department of Medical Education, Faculty of Medicine, Iran University of Medical Sciences, Tehran, Iran;; 3 Department of Philosophy of Science, Institute for Humanities and Cultural Studies, Tehran, Iran;; 4 Department of Health in Emergency and Disaster, University of Social Welfare and Rehabilitation Sciences, Tehran, Iran;; 5 Department of Clinical Science and education, Karolinska Institute, Stockholm, Sweden;

**Keywords:** Learning, Teaching hospitals, Workplace, Curriculum, Medical students

## Abstract

**Introduction:**

In medical education, particularly in residency courses, most of the training occurs in real clinical environments. Workplace-based learning profoundly affects students’ knowledge, attitudes, and practice; therefore, it should be properly planned.  Due to the extensiveness   of the clinical   environment   and   its   importance in training residents, investigating how residents learn in these environments and detecting factors that influence effectiveness will help curriculum designers to promote residents’ learning by improving their learning environment.  Therefore, our qualitative content analysis study, aimed  to  examine  the experiences and perspectives of internal and surgical residents and their attending physicians about learning in clinical settings.

**Methods:**

This qualitative content analysis study was conducted through purposeful sampling.  Semi-structured interviews were conducted with 15 internal and surgical residents and 15 of their attending physicians at educational hospitals of Tehran University of Medical Sciences.

**Results:**

The main categories explored in this study were hidden curriculum, learning resources, and learning conditions. In the context of clinical environment and under its individual culture, residents learn professionalism and learn to improve their communication skills with patients and colleagues. Because of clinical obligations such as priority of treating the patients for education or workload of the attending physicians, residents acquire most of their practical knowledge from colleagues, fellows, or follow-up patients in different learning conditions (such as: educational rounds, morning reports and outpatient clinics). They see some of their attending physicians as role models.

**Conclusion:**

Changing cultural and contextual factors is of prime importance to promote a learning-oriented environment in a clinical setting. The present findings will help curriculum planners and attending physicians to improve residents’ learning by means of appropriate workplace planning and by considering the components involved in clinical learning.

## Introduction

Postgraduate medical education residents need to learn six basic skills before graduation(1), and medical education institutes are responsible for providing an appropriate environment for the acquisition of these skills. One of the strengths of medical education, particularly in residency courses, is that most of the training occurs in real clinical environments (2).

Recent studies on workplace-based learning have revealed that this type of learning profoundly  affects  students’  knowledge,  attitudes,  and  practice,  and  thus,  it  should  be properly planned (3). At present, the effect of learning culture and context on reaching curriculum goals is receiving considerable attention (4). The importance of learning context is emphasized in most learning models. The learning context includes the learning environment (e.g., curriculum, faculty, and resident–attending relationship) and the student characteristics (e.g., motivation and ability) (5). Stable learning occurs only in supportive environments. A supportive and learning-oriented culture positively affects the competence of trained residents (4). How we think about our job and perform job-related tasks depends on our norms, values, and learning environments. The cultural web in educational environments includes norms, symbols, stories, values, rituals, and the organizational complex in clinical education and it affects identity formation of students as specialists (6).

Due to the extensiveness of the clinical environment and its importance in training residents, investigating how residents learn in these environments and detecting factors that influence effectiveness will help curriculum designers to promote residents’ learning by improving their learning environment.  Therefore, our qualitative study, aimed to examine the experiences and perspectives of internal and surgical residents and their attending physicians about learning in clinical settings. 

## Methods

In this study, the participants were selected purposefully to ensure the inclusion of 15 (10 internal and 5 surgical) residents from each year (from the first to the fourth year). Also, 15 male and female attending physicians were selected from all hospitals affiliated to Tehran University of Medical Sciences.

The data were collected through semi- structured interviews conducted by the main researcher. Depending on the participants’ choice, interviews were conducted either in the ward, or at the attending physicians’ office or at the residents’ resting room (in a quiet and comfortable atmosphere). Each resident or attending physician participated in a semi-structured interview lasting approximately 45 minutes (30-90 minutes). At first, some questions (such as: “Have you been in a morning report or educational round today? What have you done there? What have you learned?) were asked about their experiences of learning in clinical settings. Questions addressed the strategies that attending physicians used in clinical settings (morning reports, educational rounds and outpatient clinics) to support and challenge residents, and the strategies they used to provide the   residents with proper learning and feedback; then the interview process was guided based on the participants’ responses.

For this study, a qualitative method using conventional content analysis (Graneheim 2006) was considered appropriate. Therefore, the data was collected directly from the participants without any previous hypothesis. Data analysis began with data collection simultaneously. Interviews were recorded and transcribed verbatim by the main researcher. Transcriptions were reviewed and analyzed simultaneously using conventional content analysis.  

After reading the interview texts several times, the meaning units were specified, and after condensation and abstraction, they were labeled with appropriate codes.  The codes and categories were derived from an inductive process and were then conceptually ordered based on their properties and dimensions. This process continued until data saturation(7).

For data analysis, each interview was read carefully several times to determine the meaning units. Then the meaning units were abstracted through condensation to create codes; the codes were listed, reviewed and compared for similarities and differences within the interviews. Then categorization of codes was performed through reduction.

Credibility was established through prolonged engagement with data sources, triangulation of data  sources,  member  checking,  peer  debriefing  and  review  of  data  analysis  with  the supervisors.  For  dependability,  renewed  coding  of  the  interviews  was  carried  out  by colleagues who had experience in coding qualitative data. To increase the dependability and confirmability of the data, maximum variation was observed in the sampling. Also, to increase the power of data transferability, adequate description of the data was provided in the study for critical review of findings by other researchers.

This research was approved by the Research Ethics Committee of Tehran University of Medical Sciences. Informed consent was obtained from all participants and they were ensured of the anonymity of their responses and that audio files will be removed after use. 

## Results

Fifteen (11 male and 4 female) internal and surgical residents, with the mean age of 29 years, and  15  (10  male  and  5  female)  attending  physicians  with  the  mean  age  of 48  years participated in this study (Table 1).

**Table 1:** Characteristics of participants

**Occupation**	Attending physicians	**Residents**
**No.**	15	15
**Mean age**	48(32-65)	29(25-31)
**Sex**	Female:5 Male:10	Female:4 Male:11
**Specialty**	Surgery:6 Internal:9	Surgery:7 Internal:8
**Academic** **year**		Year 1:5
		Year 2:3
		Year 3:3
		Year4: 4
**Rank**	Professor=2	
	Associate professor=6	
	Assistant professor=7	

Data analysis, revealed three categories and eight sub-categories (Table 2).

**Table 2:** Data analysis results

**Categories**	**Sub-categories**	**Code**	**Meaning units**
**Hidden curriculum**	Clinical culture	Different hospital atmosphere	“Fortunately, compared to hospital … which is a very stressful place, this hospital has a good atmosphere. It is a stress-free environment where clinical education is also provided.”
		Hierarchy	“Some professors act as if we do not exist. When the hierarchy increases, and when we talk to the attending physician, he tells us to get the fellow to talk to him.”
		Different ward culture	“The residents will face a circle of information in the ward; if the system is complete and efficient, this circulation will also be effective, and the resident will benefit from it, and if not, learning does not take place properly.”
	Clinical priorities	Priority of treatment to education	“Imagine you go to the clinic and it’s crowded with a lot of patients. You must handle your patient, if you spend time on other things, the patient will be overlooked.”
		Multiple responsibilities of attending physicians	“Our job includes patient care and research as well as education. We must dedicate our energy and potential accordingly.”
**Learning conditions**	Learning in educational rounds	Theoretical discussion	“We speak about the rounds in which three others and I am were present. There is much more intimacy with attending and we can talk comfortably even about theoretical matters.”
	Learning in morning reports	Educational climate	“Sometimes there are attending physicians that really pursue your activities, what you have done last night, where you went wrong and which one of your decisions were right, and they want to teach you. You don’t really get punished if you make a mistake.”
	Learning in clinics	Limited opportunities	“If the number of patients exceeds a particular number, it will negatively affect the quality of education.”
**Learning resources**	Attending physicians	Role model	“It’s like this, just like the attending physician that evaluates me, I evaluate him as well. His movements, the way he talks to the patients, his orders for the patients, his management. And according to the image of him created in my mind and to the extent that I accept him, his teaching attracts me. It’s directly related to his performance.”
	Other medical students	Person to person learning	“Most of our training is person to person and we learn more by chief resident.”
	Patients’ follow up	Self-learning	“I think that patient follow up can show us the accuracy of our clinical reasoning. Even it is more important than our theoretical learning.”

**Hidden curriculum**

The hidden curriculum refers to the unscripted learning that occurs outside the formal curriculum. It relates to implicit values held by the clinical environment and individuals working in it. While some of this learning can be very positive, other messages can conflict with formal curriculum.  The hidden curriculum plays an important role in residents’ identity construction. Clinical culture and clinical priorities are major elements of hidden curriculum in our study.

- **Clinical culture**
  The subcategory of clinical culture consists of three major codes which refer to different ward culture, different hospital atmosphere and hierarchy.
**Different ward culture**
   It refers to patients, the emergency cases, type of treatment and diagnosis, ward atmosphere, attitude of the attending physicians toward patients and their residents which is unique in each ward. According to the opinion of most of the participants, this culture influences learning professionalism.  In this regard, one of them said,
*“Residents learn many things in wards including how to talk to the patient, and what to say and what not to say to them. The ward is a lively place….”*
**Different hospital atmosphere**
  The environment of each hospital is unique, so attending physicians-residents relationship and communication are different; this fact highly affects clinical education. Residents emphasized the important effect of the unique atmosphere of each hospital on the education they receive. In this regard one of the attending physicians said:
*“Here in this hospital teacher-student relationship is based on respect. We learn this from our teachers and teach it to our students, too.”*
**Hierarchy**
  Another important factor in hidden curriculum is the number and level of medical students and its effects on learning. In centers with fellowship training programs and in wards where fellowship students are present, their training is the first priority, and residents have less responsibility, as one participant noted,
*“Because we work in the fellowship sector, naturally we provide more education to the fellowship students as we think this is our main responsibility*.”
A resident noted, *“The problem with the subspecialty wards is that a resident may be the third person responsible for a patient; …, I do not feel that I am responsible for my patient because in the morning when I come in to visit the patient, I find that the …fellow has already visited the patient.*”
  The professors also believed that the existence of subspecialty training programs limits the chance of providing training to other students.
One of them noted, *“It seems that as the students’ competency increases, some students would be excluded from the training. It seems that the workload of the residents has decreased. In the past, the number of fellows was lower and the workload of the residents was heavier.”*
- **Clinical priorities**
**Priority of treatment to education**
  It seems that priorities and obligations in each setting significantly influence clinical education. Some of these obligations refer to the nature of clinical settings (presence of patients in there), and some refers to job regulations and other responsibilities of the attending physicians. Overall, all participants acknowledged that in clinical settings patient treatment was the first priority, and in emergencies, it became even more important. Even in providing clinical education, the priority is with those sensitive training elements which are important in saving the lives of patients. In this regard, one of the attending physicians said:
*“Our first goal is treatment and our second aim is education. We should never consider education first. I believe that the first priority is always treatment and then medical education*.”
**Multiple responsibilities of attending physicians**
  Another  subject  that  affects  clinical  education  is  the  various  responsibilities  of  the attending physicians which limit their chance to provide clinical education as they desire.
An attending physician stated, *“The education we provide depends on our workload, the number  of  faculty  members,  and  whether  they have  enough  time  to  provide  clinical education, because in addition to providing education we also have to provide treatment and conduct research. Therefore, we need to allocate our workforce to all these different tasks.*”

**Learning conditions**

This category refers to various clinical settings and is critical for learning (as participants stated). Mainly, it consists of educational rounds, morning reports and outpatient clinics.

- **Educational rounds**
  In educational rounds the way that attending physicians communicate with both patients and with residents have prime importance for learning professionalism and making a supportive climate for students and patients.
  Discussion about disease and making a decision about them are most learned topics in educational rounds
*“In rounds, usually theoretical discussions rather than practical deliberations take place”*
- **Morning reports**
  Based on the participants’ viewpoints, morning report climate is a stress free atmosphere and they can discuss scientific matters in a calm atmosphere.
*“Morning reports are good places to learn because the attending is forced to talk about the patient at the scientific level of interns and residents, and there is no much stress and anxiety”*
- **Outpatient clinics**
  There is limited opportunity for learning in outpatient clinics because there are a large number of patients and attending physicians have to provide care for them in a short period of time, so the residents cannot receive enough training.
*“The important matter is the physical environment and the large number of patients; if there are too many patients to be visited, all those involved in the treatment process including the secretaries, residents and the attending physicians become anxious and want to treat all the patients as soon as possible, and the education process will be affected*.”

**Learning sources**

Learning sources were another category that emerged from the experiences of the participants. Due to the vastness of the clinical settings and the nature of higher education in which adult learning rules are prominent, there are multiple learning resources for residents. Depends on personal favorites and usefulness, residents may choose different resources for clinical learning.

- **Attending physicians**
   In this study, residents often see the attending physicians as their role model. In fact, in addition to the  scientific  matters,  the  residents  also  learn  about  developing  a  relationship  with  the patients  and  colleagues  and  even  professional  ethics  from  the  attending physicians. Sometimes the effect of the attending physician is so profound that it also affects the choice of the educational environment.
In this regard, one resident said, *“When I observed how my professors treated their own professors, it automatically affected my attitude, or when I witnessed that my professor would greet everyone, even the orderlies of the operating room, I learned from his nice attitude*.”
However, inappropriate attitude of the attending physicians or their lack of loyalty towards the provided education has caused the residents to have inadequate training.  In this respect, a participant said *,” Some attending physicians say one thing but do another. In my opinion, if a physician talks about respecting a patient he should also be respectful towards patients. Sometimes, when we are visiting a patient, the patient is not treated respectfully by the attending physician*.”
- **Other medical students**
  Such factors as short period of the course, the large number of students at each level, the large number of patients and presence of hierarchy among the students cause the learning sources not to be limited to the attending physicians and to involve all other students at the lower and higher levels. Especially in scientific issues, physical examination and procedure, more training are done through residents at higher levels.  So a subcategory present in experiences of participants as a learning resource was other medical students.
*“In 90% of the cases, we ask our questions of the higher level residents, even those who are one level higher than us. So, I, who am in the first year of residency, ask my questions of those who are at their second year of residency. However, in 10% of the cases I can ask my questions of a fellow*.”
- **Patient follow up**
  Another source of learning is the patients themselves. Because residents are the first in line to encounter the patients, when they diagnose a patient and find that their diagnosis was accurate and once they realize that the treatment they offered was effective based on their own self-assessment, then they know that learning has occurred.
One resident said, *“Overall, one of the most important issues in surgical and other wards is follow up. When I see that what I thought about the patients’ illness and the treatment have been accurate, then I realize that I have learned about that particular illness.”*

## Discussion

This study sheds some light on the elements that, based on the opinion of the residents themselves and their attending physicians, influence residents learning in clinical environments. Residents learn most of their cognitive or practical knowledge from their colleagues or fellows or from the follow-up patients. Mostly in clinical environments, such as educational rounds, morning reports and outpatient clinics and factors like individual clinical culture, and clinical priorities are influential in their learning. 

**Hidden curriculum**

Hidden curriculum was one of the main categories in this research. Clinical culture and clinical priorities are subcategories. It was found that learning happens under the influence of individual hospital  context and culture. 

Hafferty (8-9) found that in medical education, hidden curriculum is a set of commonly held understandings, customs, rituals and taken-for-granted aspects in clinical settings. It is the influence of a series of factors at the level of culture and organizational structure which affects students and teachers in the context of formal and informal curriculum (6). Hidden curriculum is learning out of formal curriculum which is unwritten and environmental. In our study, both faculties and residents emphasized the effects of clinical culture on methods and contents of learning. They stated that the hospital general atmosphere affects some learning items including communicating with patients and colleagues and even attending physician-resident relationship. The effect of hidden curriculum on learning professionalism has also been emphasized in most researches (10-13). Professionalism is more influenced by hidden curriculum and clinical environment than formal learning and curriculum (9, 14). In clinical environments, most of the values are learned from organizational rules, assessments, documentations, stories and even speeches (15). Hidden curriculum includes socialization in medicine, as well (16).

Supervising the residents in clinical setting, developing a better relationship with them and providing them with more feedback are important factors in clinical learning (17). In this research, fellowship programs and presence of fellows in clinical settings were important clinical organizational factors which caused residents to have few responsibilities over the patients, and receive few supervision and feedback from the attending physicians. This finding is in accordance with studies which found that clinical organizational structure affects residents’ skills and reduces them (18).

Perceived priorities and obligations in clinical settings were another category that affected clinical learning.  Almost all attending physicians and residents emphasized the priority of treating patients over training residents. Considering this approach, clinical learning will be less influenced by the following factors: the time limitation in visiting the patients, the large number of patients that need to be visited (in outpatient clinics) or the presence of high risk patients (19).

Those   attending physicians  who  were   interviewed   said   that  abundance   of   their responsibilities was one of the factors which affected residents’ learning. Some studies conducted on the responsibilities of attending physicians also revealed that they have multiple responsibilities in clinical settings and spend few hours to supervise residents (20).

**Learning conditions**

Morning-reports, educational rounds and out-patient clinics are most mentioned learning environments in this research. Recent investigations about learning environments for achieving core competencies in medical education, declared that educational leadership have to pay more attention to the quality of learning in these environments (21). In our research, too many patients and limited time sometimes causes poor quality of teaching and learning in outpatient clinics. Lack of time still remains important in educational rounds and morning reports, but there are more learning opportunities in case of the presence a stress-free environment.

**Learning resources**

Clinical learning resources are another category. Attending physicians, other medical students and patient follow-up are the most important resources that participants mentioned. Residents see the attending physicians as role models in clinical education, and this was in line with the finding of a study by Goldie, et al. (2015) that acknowledged effective attending physicians as good role models and emphasized the importance of the role of their non-cognitive characteristics such as developing interpersonal relationships (19).   In this research, many residents pointed to learning professionalism of their attending physicians which is in line with other studies (10, 22). Some residents also recognized the important role of attending physicians in their placement and this is also in agreement with that of Goftman and Regher studies (17, 22).

It is common to learn from those residents at higher academic levels or even from colleagues in clinical settings. Medical students often see residents as mentors who have a supportive role while they acknowledge attending physicians as role models (23). This is even bolder in surgical wards as medical students see residents as their teacher more than their attending physicians. It is said that medical students spend most of their time in surgical wards with surgical residents (24); this is the case for lower academic level residents, too. Furthermore, because of   residents’ position in clinical settings and because of their closeness to medical students, it would be highly beneficial for both patients and medical students if residents receive training to become clinical teachers (25).

One of the residents’ learning methods was to follow up patients. This is one of the self- directed methods of learning which has also been emphasized in other researches (24-25).

## Conclusion

In order to achieve ACGME core competencies in residency education, improving quality of clinical learning is important. It seems that learning in clinical settings is highly dependent on clinical culture and context. So promoting residents’ clinical learning is dependent on improving its own culture and organization. Changing learning conditions is of prime importance to pave the way to develop learning–oriented environments in clinical settings. Nowadays, planning for learning in work-based places has been highly recommended in many researches. In this regard, curriculum planners have to heighten the quality of learning resources in clinical settings. The findings of this study help curriculum planners and attending physicians to improve residents’ learning by means of appropriate planning and considering the components involved in clinical learning. 

## Footnotes

Authors contribution: Mehrnaz- Geranmayeh developed the study design, conducted the interviews and analysis, ensured trustworthiness, and drafted the manuscript. Alireza Esteghamati and Hamidreza Baradaran as the supervisors participated in the study design, supervised the codes and data analysis process, and revised the manuscripts. Hamidreza Khankeh advised the codes and data analysis process and revised the manuscript.
